# Supplementary material for: Identification of photoperiod-induced specific miRNAs in the adrenal glands of Sunite sheep (Ovis aries)
Source: Front Vet Sci. 2022 Jul 22;9:888207. doi: 10.3389/fvets.2022.888207 (PMC9354845; doi:10.3389/fvets.2022.888207)
Supplement: Supplementary file 1 [file Data_Sheet_1.ZIP › Supplementary file/miRDeep2 file.zip!/miRDeep2 file/Know_Structure/LP42A2/oar-mir-432.pdf]

miRBase precursor : oar-mir-432
Total read count : 1646
oar-miR-432 read count : 1636
remaining reads : 10

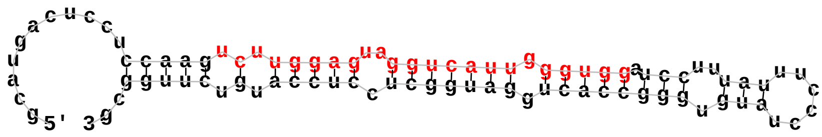

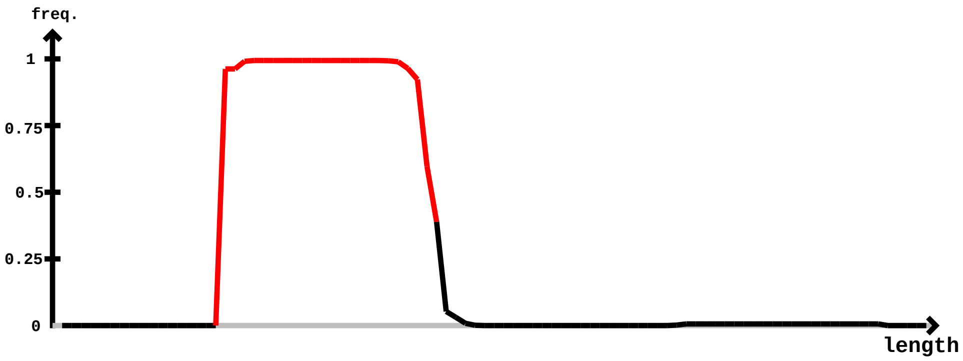


```
                oar-miR-432
5'- gcaugacuccuccaagucuuggaguaggucauuggguggauccuuuauuucccuaugugggccacuggauggcuccuccaugucuuggcg   -3'    exp
    ...........(((((.(.(((((..(((((((.(((((.(((..(((......))).)))))))).))))))).))))).).)))))..    reads    mm    sample
    ................ucuuggaguaggucauu.........................................................    2        0     seq
    ................ucuuggaguaggucauug........................................................    6        0     seq
    ................ucuuggaguaggucauugg.......................................................    41       0     seq
    ................ucuuggaguaggucauuggg......................................................    68       0     seq
    ................ucuuggaguaggucauugggu.....................................................    536      0     seq
    ................ucuuggaguaggucauugggug....................................................    342      0     seq
    ................ucuuggaguaggucauuggguggg..................................................    549      0     seq
    ................ucuuggaguaggucauuggguggga.................................................    32       0     seq
    ................ucuuggaguaggucauugggugggau................................................    7        0     seq
    ................ucuuggaguaggucauugggugggauc...............................................    1        0     seq
    ..................uuggaguaggucauugggug....................................................    1        0     seq
    ..................uuggaguaggucauuggguggg..................................................    4        0     seq
    ..................uuggaguaggucauuggguggga.................................................    4        0     seq
    ..................uuggaguaggucauugggugggau................................................    27       0     seq
    ..................uuggaguaggucauugggugggauc...............................................    9        0     seq
    ..................uuggaguaggucauugggugggaucc..............................................    2        0     seq
    ...................uggaguaggucauugggugggau................................................    3        0     seq
    ...................uggaguaggucauugggugggauc...............................................    2        0     seq
    ...............................................................acuggauggcuccuccaugucu.....    3        0     seq
    ................................................................cuggauggcuccuccaugucu.....    7        0     seq
```
